# Implementation of integrated care for type 2 diabetes: a protocol for mixed methods research

**DOI:** 10.5334/ijic.1516

**Published:** 2014-12-15

**Authors:** Loraine Busetto, Katrien Ger Luijkx, Hubertus Johannes Maria Vrijhoef

**Affiliations:** Department of TRANZO, Faculty of Social and Behavioural Sciences, Tilburg University, PO Box 90153, 5000 LE Tilburg, The Netherlands; Elderly Care, Department of TRANZO, Faculty of Social and Behavioural Sciences, Tilburg University, PO Box 90153, 5000 LE Tilburg, The Netherlands; Health Systems & Policy, National University Singapore; Chronic Care, Tilburg University, PO Box 90153, 5000 LE Tilburg, The Netherlands

**Keywords:** integrated care, type 2 diabetes, implementation, disease management, chronic care model, mixed methods

## Abstract

**Introduction:**

While integrated care for diabetes mellitus type 2 has achieved good results in terms of intermediate clinical and process outcomes, the evidence-based knowledge on its implementation is scarce, and insights generalisable to other settings therefore remain limited.

**Objective:**

This study protocol provides a description of the design and methodology of a mixed methods study on the implementation of integrated care for type 2 diabetes. The aim of the proposed research is to investigate the mechanisms by which and the context in which integrated care for type 2 diabetes has been implemented, which outcomes have been achieved and how the context and mechanisms have affected the outcomes.

**Methods:**

This article describes a convergent parallel mixed methods research design, including a systematic literature review on the implementation of integrated care for type 2 diabetes as well as a case study on two Dutch best practices on integrated care for type 2 diabetes.

**Discussion:**

The implementation of integrated care for diabetes type 2 is an under-researched area. Insights from this study could be applied to other settings as well as other chronic conditions to strengthen the evidence on the implementation of integrated care.

## Introduction

Diabetes mellitus type 2 has become a widespread problem in many Western societies. In 2010, the global diabetes prevalence among people aged 20–79 years was estimated at 6.4%; in the European Union and the Netherlands, prevalence in similar age groups was respectively 6% and 7% in the same year [1–3]. Due to these high prevalence rates, diabetes has a major impact on society in terms of the economic costs incurred by diabetes patients. Research indicates that 12% of global health expenditure was spent on diabetes in 2010 [4]. European Union countries spent approximately 10% of their total health expenditure on diabetes in 2010 [2, 4] and in the Netherlands, 2–9% of total health expenditure was spent on diabetes care in 2010/2011, depending on the registration of co-morbidity and the extent to which diabetes-related complications are considered in the estimations [4, 5].

Previous systematic reviews have shown that integrated approaches to diabetes care can yield improvements in care delivery process as well as intermediate clinical outcome indicators. Benefits have been found for process indicators such as screening for retinopathy [6–8], foot lesions [6–8], periphal neuropathy [7], proteinuria [7], and monitoring of lipid concentrations [7] and glycated haemoglobin [7], as well as intermediate clinical outcome indicators such as glycated haemoglobin [6, 8–10], blood pressure [8, 11] and blood lipid control [10, 11]. In addition, previous systematic reviews have demonstrated the added value of integrated chronic care in terms of economic benefits [12]. However, other reviews have shown no (significant) impact on the above process and outcome indicators [7, 13] or have disputed the clinical relevance of statistically significant findings [10].

There is still a lack of evidence regarding the question which integrated care programmes are effective in which circumstances. Despite the fact that several previous studies have pointed out the importance of studying implementation [14–16], all of the above shows that there is a disproportionate emphasis on the goal-achievement and effectiveness of integrated care for type 2 diabetes rather than the intricacy of the implementation. By stripping away all confounding factors so as to be able to study the intervention's pure effect on the outcome, researchers run the risk of proclaiming programme failures prematurely as well as being blinded to the actual determinants of success or failure [17].

This article describes the design of a mixed methods study on the implementation of integrated diabetes care, combining a literature review of international integrated diabetes care with a case study on two Dutch best practices on integrated care for type 2 diabetes. The aim of the proposed research is to identify the different contexts in which and mechanisms by which integrated care for type 2 diabetes has been implemented, to report the outcomes achieved and to investigate how the contexts and mechanisms have affected these outcomes. This study is part of Project INTEGRATE on ‘Benchmarking Integrated Care in Chronic and Age-related Conditions in Europe’, financed by the European Commission (project reference 305821). Project INTEGRATE aims to investigate the leadership, management and delivery of integrated care to help European care systems responding to the challenges of an ageing population and the increasing number of people living with chronic conditions [18, 19].

The proposed research focuses on the following four overall research questions:

- By which mechanisms has integrated care for type 2 diabetes been implemented?
- In which contexts has integrated care for type 2 diabetes been implemented?
- What were the outcomes of integrated care for type 2 diabetes?
- How have the contexts and mechanisms by which integrated care for type 2 diabetes has been implemented affected its outcomes?

## Methods

### Research Design

A mixed methods design will be used for this study as this is the most appropriate research design for studying the implementation process as well as the outcomes of integrated care.

As Pawson and Tilley point out, classical methodologies usually focus on observations at two specific points in time, namely before the intervention and after the intervention [20]. In order to increase the ability to attribute the differences observed post-intervention to the intervention itself (instead of ‘third variables’), most factors expected to have a confounding effect on the causal relationship are stripped away. However, for complex interventions, which can be seen as ‘dynamic complex systems thrust amidst complex systems’ [21], it is often precisely those factors left out of the equation which hold the most valuable information [17, 20].

To avoid this methodological pitfall, several qualitative methodologies will be used and combined with quantitative methods, which, according to Berwick, is an approach superior to the more classical methodologies such as randomised controlled trials [17]. We decided to use a convergent parallel mixed methods design which involves concurrent implementation of the qualitative and quantitative research strands, equal prioritisation of the quantitative and qualitative methods, independent analysis of both strands with traditional methods and merging of strands during overall interpretation [22].

Specifically, the design includes a systematic literature review and a case study to be qualitatively analysed with an explicit focus on context, mechanisms and outcomes. Moreover, local wisdom will be emphasised by actively involving local stakeholders instead of excluding them for fear of bias [17]. This will enable the researchers to access the stakeholders’ insights into the details of the implementation that might otherwise remain hidden from their view. In addition, for the case study, quantitative patient outcome data will be collected and analysed. After independent analyses, the qualitative and quantitative results will be combined for overall interpretation.

### Operationalisation

#### Integrated care

In order to determine which interventions can be considered integrated care, it is important to operationalise what we mean by integrated care. Given the quasi-universal acceptance of Wagner's chronic care model and its widespread use throughout the literature [23–25], we decided to link our understanding of integrated care to the chronic care model. In line with previous research, it was decided that if an intervention targets at least two of the four core chronic care model components, the intervention is to be considered integrated care [8, 26, 27].

When assessing whether a study is indeed concerned with integrated care, it is important to ensure that all researchers apply the same understanding of the components. Hence, it was decided to operationalise the four chronic care model components to be used for the review. This operationalisation is largely based on the checklist used in the ‘Developing and Validating Disease Management Evaluation Methods for European Health Care Systems’ project [28] and complemented by other definitions and examples of the chronic care model components in the literature [29–32]. Table 1 depicts the operationalisation of the chronic care model to be used in the literature review.

#### Implementation

By ‘implementation’, we mean the bringing into practice of a model for change, which is always implemented by certain mechanisms and in a certain context. The specific terminology of ‘mechanism’ and ‘context’ used in this study is derived from Pawson and Tilley‘s work on realistic evaluation [20]. Their main claim is that it is both the context in which an intervention is implemented (including the organisational, financial, political, technological and human constraints) as well as the mechanisms by which it is implemented (including assumptions of how change can be achieved) that will affect the outcomes that can be achieved by the intervention [20, 33]. This means that instead of asking whether an intervention worked, the purpose of realist enquiry is to identify the mechanisms and context and to find out which mechanisms work in which context to achieve which outcomes [20, 21, 33].

##### Mechanism

By ‘mechanism’ we mean the different types of integrated care for type 2 diabetes distinguished into ‘programmes’ and ‘interventions’. By ‘programme’ we mean a set of at least two interventions whose combined implementation is intended to lead to the achievement of a certain goal, often an improvement in the quality of care. By ‘intervention’ we mean the tangible actions that, combined, constitute a programme.

##### Context

The context of implementation consists of implementation strategies and an implementation process. By ‘implementation strategies’ we mean information and plans concerning what to do to facilitate and improve the working of the change model in practice, explicitly formulated prior to the realisation of the model for change in practice. By implementation process, we mean the process of ‘social change’ triggered by the mechanisms, which inherently, is sensitive to a multitude of context factors that impact on this process [17]. We describe the implementation process through the description of those factors encountered during the implementation process and explicitly identified by the stakeholders as barriers or facilitators to the implementation of the integrated diabetes care programme or intervention.

##### Outcomes

By ‘outcomes’ we mean the intended and unintended consequences triggered by mechanism and context, including both process outcome measures and intermediate clinical outcome measures. Process outcome measures include (but are not limited to): frequency of measurements of HbA1c/A1C, blood pressure, and lipids, frequency of patient consultations, recommendation to take aspirin, dilated retinal examinations, urine tests, statin therapy prescription and receipt of influenza vaccination. Intermediate clinical outcome measures include (but are not limited to): HbA1c/A1C, blood pressure and LDL values.

### Literature Review

The literature review aims to provide answers to the research questions from an international perspective.

For the first research question, the integrated care programmes and interventions identified through the systematic literature search will be described in detail and classified according to the chronic care model as operationalised by the authors (see Table 1). For the second research question, qualitative analyses will be performed to summarise the strategies for as well as barriers and facilitators to the implementation of integrated care for type 2 diabetes, as identified in the literature. For the third research question, qualitative analysis will yield an overview of the outcomes of the integrated diabetes care programmes and interventions described in the literature. Finally, it will be investigated to what extent and in what way the implementation strategies and process affected the outcomes.

#### Search strategy

In order to find relevant articles, four groups of search terms will be created: (1) search terms related to the health condition, (2) search terms describing the type of intervention, (3) search terms related to the four chronic care model components and (4) the search term ‘implementation’ (see Table 2). The four groups of search terms will be connected with Bolean operators in such a way that articles concerned with diabetes and an integrated care type intervention (or combinations of two out of the four chronic care model components) and implementation will be retrieved. The databases Pubmed/Medline and Cochrane will be searched for eligible articles.

#### Selection

A total of three screening rounds will be performed based on readings of titles, abstracts and full texts. In each round, articles will be included based on the following inclusion criteria: (1) published between 2003 and 2013; (2) concerns integrated care; (3) focuses on type 2 diabetes or focusses on type 2 diabetes and one or more additional condition(s) and reports results for each condition separately.

Articles written in a language other than English or one of Project INTEGRATE's case study languages (German, Dutch, Spanish and Swedish) will be excluded. Articles with a target population consisting only of children, adolescents, prisoners or homeless persons will be excluded as they do not match the target population of the two Dutch case studies. Articles not concerning empirical research analysing the implementation of interventions will be excluded. Additionally, systematic reviews and meta-analyses will be excluded because these types of studies report results on a rather abstract level of evidence which might mask insights that are relevant for this implementation-focussed type of study. In all exclusion rounds, articles can be excluded for more than one reason. When in doubt or when the title or abstract does not give enough information to base a decision on, articles remain included.

The screenings will be performed by three independent researchers. To ensure a homogeneous selection, a checklist based on the above operationalisation of the chronic care model and the previously mentioned in- and exclusion criteria will be used by all researchers. After this, the results will be discussed in pairs in order to create agreement on the interpretation of the criteria. When in doubt or disagreement, discussions between the researchers will take place until consensus is achieved.

#### Data analysis

After the article selection, the included studies will be analysed. Data extraction and quality assessment for each article will be performed independently by three researchers using a standardised data extraction form to ensure uniformity. The following information will be extracted from the articles: general information (including author, year of publication and title), methodological information (including data collection methods, type of data collected, setting or context of data collection, follow-up period, population and participants, researcher's influence, data analysis, research questions and/or article objective, study limitations), information on the integrated care programme or intervention (including the name of the programme or intervention, its purpose and the specific interventions of which the programme consists), implementation strategies, barriers, facilitators and outcomes of the integrated care programme or intervention. Based on this information, the articles' quality will be assessed by using the 2011 version of mixed methods appraisal tool [34, 35]. The mixed methods appraisal tool is a unified tool that can be used for the simultaneous quality assessment of qualitative, quantitative and mixed methods studies [34]. Despite its relative novelty, the mixed methods appraisal tool has already been used as a comprehensive quality assessment tool in various systematic reviews in the health sciences [36–38]. See Table 3 for a tabular overview of the quality aspects to be assessed per type of study.

After the extraction and assessment, the researchers will compare and discuss the forms until disagreements can be resolved by consensus.

Additionally, the implementation model by Grol and Wensing will be used for the categorisation of the context factors identified in the literature review [39]. According to this model, barriers to and incentives for change occur at six different levels of health care, namely innovation, individual professional, patient, social context, organisational context, and economic and political context [39]. Grol and Wensing's model has been used for the categorisation of barriers and facilitators to integrated care for diabetes type 2 in several previous studies [16, 40, 41].

The results from the literature review will be used as a context for the insights gained from the case study and will enable the identification of differences and commonalities between the international literature and the Dutch case.

### Case Study

In order to answer the research questions from the Dutch perspective, a case study on Dutch integrated diabetes care will be conducted at two separate case sites.

#### Case selection

Two care groups will be invited to participate as best practice case sites in the case study research.

Care groups are legal entities with their own managerial and administrative staff, often (co-) owned by general practitioners, that cooperate with a variety of health care providers involved in the provision of chronic care.

The decision to focus on national best practices is based on the assumption that identifying success factors encountered by the frontrunners of diabetes care innovation will generate meaningful lessons for those that are now encountering or will still have to encounter similar barriers in the future. Moreover, focusing on best practices will generate an important potential for learning by other Dutch care groups, and given the Netherlands’ long experience in integrated care and status as a pioneer [42, 43], also for other European and non-European countries.

Despite the popularity and wide-spread use of best practices research, its use in scientific research is controversial, most notably due to the limited external validity of this case-based approach [44, 45]. Therefore, it should be noted that the authors define best practices as ‘best practices for the process of planning for most appropriate interventions for the setting and population’ [44]. This definition entails that the envisaged outcome of best practices is not a generalisable plan but a generalisable process for planning [44].

The following criteria will be pivotal in the selection of the care groups: nomination as national best practices by leading health research institutions, participation in previous (diabetes) research; involvement in care innovation pilots such as those recently selected by the Dutch Minister of Health; and Welfare and Sport to be closely followed in the upcoming years [46].

#### Data collection

Data from the two case sites will be collected by means of a document review, semi-structured interviews and routine health care data.

##### Document review

The documents will be provided by the two case sites' respective contact persons. Initially, the interviewers will request documents that cover the whole cycle of implementation, from the initial idea via planning, implementation, evaluation and adaptations to the current state of affairs. At a later stage, additional documents will be requested for those phases not adequately covered by the initial set of documents. The documents to be collected include regional policy documents, performance evaluation reports, annual reports, focus group reports, improvement plans, educational programmes and other documentation. The main purpose of the document review is as preparation for the interviews, to serve as illustration and for the triangulation of the interview results.

##### Interviews

In addition to the document study and the collection of routine health care data, 25 interviews will be conducted for each case site. Interviews will be chosen as main method of data collection because their purpose is to gain an overview of the variations in perspectives and opinions and the circumstances that play a role [47]. In addition, interviews are the preferred method of data collection when the research question refers to opinions and experiences (as opposed to actions) which only the interviewee can access [48], which is applicable to this case, especially regarding the barriers and facilitators encountered during the implementation process.

Of the 25 interviews to be conducted per care group (50 in total), 10 will be held with diabetes patients and the other 15 with care group directors, managers and staff as well as health care providers involved in the organisation and delivery of integrated diabetes care, including general practitioners, internists, diabetes nurse specialists, practice nurses, dieticians, pharmacists, optometrists, podiatrists and pedicurists. Precisely which persons and professions will be approached, will be decided in consultation with the care group contact persons. We expect that a heterogeneous sample including patients as well as all relevant health professions and care group staff involved in diabetes care will create as complete a picture as possible, consisting of many diverse perspectives, experiences and opinions.

Interviewees will be requested to sign an informed consent form, indicating that he or she has read the information leaflet and had the opportunity to ask questions, that he or she understands that the participation in the research is on a voluntary basis and can be revoked at any time, that he or she agrees to participate in the research and with the interview being audio-taped. All interviews will be audio-recorded and transcribed.

During the interviews, the interviewers will use a topic list to help the interviewer steer the conversation via predefined topics and initial questions [47]. The topic list for the health professionals will focus on the areas of integrated care in general and in the interviewee's institution, implementation of integrated care, information technology, finance and sustainability of integrated care.

As previous research with patients suffering from chronic disease has shown the importance of giving patients the opportunity to tell their illness narratives [49–51], the patients’ topic list will focus on the patients’ personal experiences with their disease, their knowledge and experiences about integrated care and the care group they are a part of, the barriers and facilitators they encountered to their care as well as the health outcomes they achieved and how the former may have affected the latter. While establishing rapport between the interviewer and interviewee is important in all individual interviews, it is especially so for the more vulnerable target groups such as (elderly) patients. Therefore, the four stages of building rapport, namely apprehension, exploration, co-operation and participation, will be given special emphasis in the patient interviews [52].

In both cases, the number and nature of the sub-questions can vary, as can the pre-defined topics if considered necessary during the research process [47]. Additional and follow-up interviews will be conducted until saturation is achieved regarding the scope and the detail of the research.

To assure the quality of the interviews conducted, a member check will be performed by sending a one page summary of each interview to the interviewees who will then be asked whether this summary reflects their point of view and statements made during the interview. In case of negative feedback by the interviewee, a follow-up interview will be scheduled for clarification.

##### Routine health care data

To measure health outcomes, diabetes type 2 patients’ routine health care data will be collected. These will be provided by the care groups participating in the case study. They have access to the data from all diabetes type 2 patients in treatment by general practitioners who are members of the care group as the collection of these data in a common information technology system is a requirement for membership of the care group. Data will be collected for the period from 2008 (start of systematic data collection by the care groups via the electronic medical record) to 2014 (start of data collection by the researchers). The collected data include intermediate clinical outcome measures (e.g. glycated haemoglobin, low-density lipoprotein, systolic blood pressure and body mass index) as well as process outcome measures (measurements of glycated haemoglobin, low-density lipoprotein, systolic blood pressure and body mass index) [31, 53].

#### Data analysis

For the analysis of the interviews, the audio-recordings will be transcribed verbatim and coded independently by two researchers. Given the inherently iterative nature of qualitative research [54], the coding of the interview transcripts will be performed in three phases, namely open coding, axial coding and selective coding [54, 55].

In the open coding phase, which is characterised by its exploratory nature [54], two researchers will label fragments of the text material with descriptive as well as interpretive codes based on the interviewees’ own wording (in vivo codes) and prominent concepts from the literature study described above (constructed codes) [56, 57]. The second phase, axial coding, involves finding and describing important concepts and making a distinction between the more or less relevant codes so as to reduce the amount of material [55, 57]. In the selective coding phase, the researchers will start searching for explanations of the phenomena that were found as well as the relationships between different categories [55, 58].

All coding activities described above will be performed independently by two researchers. This will help to limit bias and assure the quality of the analysis as well as enable the development of a well-structured coding system [55]. In addition, it helps to improve the validity and objectivity of the results [58]. Disagreement will be resolved by consensus through bilateral discussions. All coding and analysis activities will be performed in Atlas.ti 6. Furthermore, as for the literature review, also for the case study, the implementation model by Grol and Wensing will be used for the categorisation of the context factors identified [39].

For the quantitative data, statistical analyses will be performed in SPSS 19. Multi-level analyses will be performed to describe the development of process and intermediate patient outcomes over time at baseline (t0) and yearly intervals until 2014 (t6). Moreover, the intermediate and process outcomes for each care group will be compared using analysis of variance. Sex, age, diabetes type and diabetes duration will be included as potential confounders.

As mentioned above, special emphasis will be put on the integration of qualitative and quantitative data, by comparing quantitative clinical data to qualitative patient stories and explaining how they relate to each other. Moreover, the results from the analysis of the interviews and document study will be triangulated with the results from the literature review. This entails that the results from the literature review will provide a context for interpretation of the case study results by providing the basis for the coding process of the interviews. This will enable us to give a combined answer to the same research questions, based on different sources of knowledge.

## Discussion

This paper presents the design of a mixed methods study to be conducted on the implementation of integrated care for type 2 diabetes. The chosen combination of methods of data collection and analysis will enable a thorough study of the mechanisms by which and contexts in which integrated care for type 2 diabetes has been implemented, which outcomes have been achieved and how the former affected the latter. Especially the combination of the international literature review and the national case study will provide added value through the triangulation of results and the provision of an international embedding of national research.

An important strength of this article is its grounding in different conceptual models, including Pawson and Tilley's realistic evaluation framework, the chronic care model and the implementation model, which all adopt a holistic approach to implementation analysis. While realistic evaluation makes it possible to study the links between the intervention, its implementation and outcomes achieved, the six chronic care model components and implementation model levels, respectively, make it possible to capture the whole range of inner local/organisational factors as well as outer national/regulatory factors. This is especially important given the Netherlands’ national set-up of integrated care via care groups and bundled-payment contracts which are implemented differently per local context. The authors believe that the interviewees to be selected for this study will be able to identify and elaborate on the links between the national/regulatory and local/organisational factors and connect them to the likelihood of a successful implementation in practice.

There are also some limitations to this prospective study which need to be taken into consideration. First, the decision to link the definition of integrated care to the chronic care model might blind the researchers to aspects of care integration that are not described by the chronic care model. The choice of the chronic care model, however, is based on its acceptance and use in the international literature as well as national practice, assuming that this indicates the model's scientific and societal relevance and applicability.

The second limitation concerns the decision to focus the literature search only on the four core elements of the chronic care model. By not actively searching for health system and community interventions, the search might miss publications of potential added value to the research. However, given the study's explicit focus on the implementation of programmes and interventions, the researchers feel the necessity to limit the search to the most tangible of interventions. It is likely that the programmes identified through the literature search will often also include aspects of the health system and community components even if they are not actively searched for.

The third limitation lies in the study's focus on best practices. Despite the many advantages this entails, focusing on best practices only means that the results from the prospective study will not provide any information about average Dutch diabetes care. By not including other care groups in the research, it will also not be possible to report the exact aspects in which the two selected case sites differ from other Dutch care groups and whether these differences might limit the external validity as well as applicability of the results to other care groups. The literature review, however, applies an international perspective and balances the focused perspective of the case study.

## Conclusion

Systematic investigation of the implementation of integrated care is insufficiently highlighted. This research fills the gap in knowledge on how to best implement integrated care for type 2 diabetes, taking into account the specific mechanisms and contexts that affect the outcomes to be achieved. In doing so, this study will form the basis of tangible recommendations to health practitioners, managers and policy makers as to what can or should be implemented in which circumstances and what the expected results can be. Insights from this study could be applied to other settings as well as other chronic conditions to strengthen the evidence on the implementation of integrated care.

## Figures and Tables

**Table 1.**
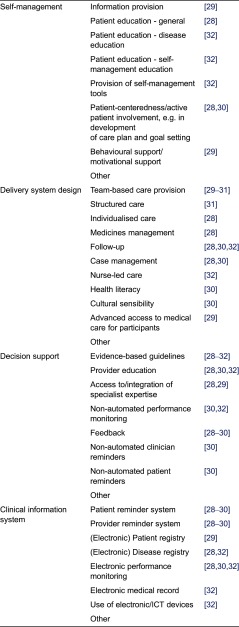
Operationalisation of the four core chronic care model components

**Table 2.**
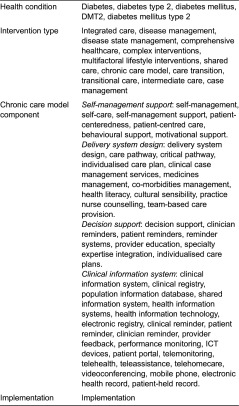
Four groups of search terms

**Table 3.**
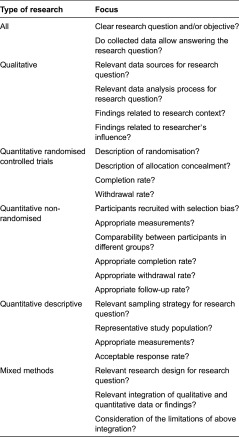
Tabular overview of the mixed methods appraisal tool [34]
